# Supplementary material for: Diagnosis of Schizophrenia and Its Subtypes Using MRI and Machine Learning
Source: Brain Behav. 2024 Dec 31;15(1):e70219. doi: 10.1002/brb3.70219 (PMC11688118; doi:10.1002/brb3.70219)
Supplement: Supplementary file 1 — Supporting Information [file BRB3-15-e70219-s001.docx]

**Table S1** Accuracy (mean±std) of machine learning models applying different features and combining with feature selection methods for classification of healthy and schizophrenia groups.

| Model  Features | kNN (k = 5) | SVM (Linear) | SVM (Polynomial) | LDA | LR | RF | NB |
| --- | --- | --- | --- | --- | --- | --- | --- |
| Subcortical Volume | 0.60±0.03 | 0.70±0.02 | 0.50±0.00 | 0.63±0.02 | 0.59±0.02 | 0.69±0.03 | 0.64±0.00 |
| Cortical thickness | 0.58±0.02 | 0.59±0.03 | 0.53±0.01 | 0.53±0.03 | 0.51±0.03 | 0.66±0.03 | 0.69±0.01 |
| Graph measures | 0.61±0.02 | 0.46±0.03 | 0.53±0.01 | 0.53±0.02 | 0.53±0.04 | 0.58±0.02 | 0.61±0.00 |
| Volume + Graph | 0.63±0.04 | 0.57±0.02 | 0.49±0.00 | 0.66±0.01 | 0.52±0.21 | 0.61±0.03 | 0.65±0.01 |
| Thickness + Graph | 0.64±0.02 | 0.54±0.03 | 0.53±0.01 | 0.56±0.02 | 0.54±0.26 | 0.62±0.02 | 0.57±0.02 |
| Volume +Thickness + Graph | 0.66±0.02 | 0.60±0.02 | 0.42±0.01 | 0.63±0.01 | 0.51±0.02 | 0.67±0.03 | 0.64±0.02 |
| SFS (5 features) | 0.66±0.02 | 0.67±0.02 | 0.60±0.02 | 0.69±0.01 | 0.60±0.17 | 0.61±0.04 | 0.66±0.00 |
| MRMR  (Number of features) | 0.79±0.04 ^1^  (12) | 0.77±0.03  (11) | 0.73±0.03  (19) | 0.76±0.07  (6) | 0.63±0.02  (60) | 0.79±0.03  (22) | 0.75±0.02  (23) |
| NCA  (Number of features) | 0.68±0.04  (38) | 0.70±0.04  (34) | 0.68±0.03  (39) | 0.68±0.48  (9) | 0.58±0.01  (9) | 0.73±0.05  (44) | 0.64±0.02  (11) |

^1^ Highest accuracy

**Table S2** Accuracy (mean±std) of machine learning models applying different features and combining with feature selection methods for classification of healthy and subtypes of schizophrenia.

| Model  Features | kNN (k = 5) | SVM (Linear) | SVM (polynomial) | LDA | LR | RF | | NB | |
| --- | --- | --- | --- | --- | --- | --- | --- | --- | --- |
| Subcortical Volume | 0.28±0.03 | 0.34±0.04 | 0.38±0.03 | 0.29±0.03 | 0.44±0.00 | | 0.36±0.04 | | 0.35±0.02 |
| Cortical thickness | 0.46±0.02 | 0.27±0.04 | 0.44±0.01 | 0.34±0.02 | 0.41±0.01 | | 0.36±0.03 | | 0.31±0.01 |
| Graph measures | 0.41±0.03 | 0.36±0.02 | 0.32±0.03 | 0.35±0.01 | 0.43±0.00 | | 0.42±0.01 | | 0.35±0.02 |
| Volume + Graph | 0.43±0.03 | 0.37±0.02 | 0.36±0.04 | 0.42±0.01 | 0.43±0.01 | | 0.37±0.03 | | 0.32±0.02 |
| Thickness + Graph | 0.43±0.03 | 0.40±0.03 | 0.31±0.04 | 0.43±0.02 | 0.41±0.01 | | 0.38±0.05 | | 0.32±0.02 |
| Volume +Thickness + Graph | 0.36±0.01 | 0.43±0.03 | 0.38±0.03 | 0.47±0.01 | 0.44±0.00 | | 0.40±0.03 | | 0.35±0.02 |
| SFS (6 features) | 0.36±0.03 | 0.36±0.03 | 0.32±0.03 | 0.40±0.02 | 0.43±0.00 | | 0.30±0.03 | | 0.32±0.03 |
| MRMR (Number of features) | 0.59±0.04  (32) | 0.64±0.02 ^1^  (62) | 0.51±0.04  (70) | 0.41±0.04  (1) | 0.48±0.02  (34) | | 0.54±0.04  (55) | | 0.31±0.02  (1) |
| NCA (Number of features) | 0.42±0.03  (7) | 0.45±0.04  (21) | 0.44±0.05  (97) | 0.43±0.04  (1) | 0.44±0.01  (10) | | 0.51±0.02  (1) | | 0.41±0.05  (1) |

^1^ Highest accuracy

**Machine Learning methods in details**

**1- Support Vector Machine (SVM)**

SVM is a powerful supervised learning algorithm used primarily for classificationو, regression and outlier detection. The core idea behind SVM is to find the optimal hyperplane that best separates data points of different classes in high-dimensional space. This hyperplane is positioned such that it maximizes the margin, which is the distance between the hyperplane and the nearest data points from each class, known as support vectors. Mathematically, the decision boundary can be defined by the equation:

$$w.x+b=0$$

where $w$ represents the weight vector perpendicular to the hyperplane, $x$ denotes the input feature vector, and $b$ is the bias term. The margin is defined as:

$$Margin= \frac{2}{\left\| w \right\|}$$

The SVM algorithm employs various kernel functions including linear, polynomial, Gaussian, and sigmoid to transform input data into higher dimensions, allowing for effective separation even in non-linear cases. The kernel enables this transformation without explicitly computing the coordinates in the higher-dimensional space.

**2- k-Nearest Neighbors (kNN)**

kNN algorithm is another supervised learning technique for classification and regression tasks based on the principle of proximity. It operates by identifying the $k$ closest training examples to a new data point, utilizing a distance metric such as Euclidean distance, defined mathematically as:

$$d\left( x,y \right)= \sqrt{\sum_{i=1}^{n} {(x_{i}-y_{i})}^{2}}$$

where $x$ and $y$ are two data points in an $n$-dimensional space. For classification purpose, kNN assigns the class label that is the most frequent among the $k$ nearest neighbors, while for regression tasks, it predicts the value based on the average of these neighbors. The choice of $k$ is critical; a smaller $k$ can lead to noise sensitivity and overfitting, while larger one may smooth out class distinctions. The algorithm’s simplicity and effectiveness make it popular in various applications.

**3- Linear Discriminant Analysis (LDA)**

LDA is a statistical method used for classification and dimensionality reduction. It is particularly effective when the classes are normally distributed with equal covariance matrices. The primary goal of LDA is to find a linear combination of features that best separates two or more classes. This is achieved by maximizing the ratio of between-class variance to within-class variance, which can be mathematically expressed as:

$$J\left( w \right)= \frac{w^{T}S_{B}w}{w^{T}S_{W}w}$$

where $S_{B}$ represents the between-class scatter matrix and $S_{W}$ denotes the within-class scatter matrix:

$$S_{B}=\sum_{i=1}^{n} n_{i}(\mu_{i}- \mu){(\mu_{i}-\mu)}^{T}$$

$$S_{W}=\sum_{i=1}^{n} \sum_{x\in D_{i}} ({x-\mu}_{i}){({x-\mu}_{i})}^{T}$$

where $n$ is the number of classes, $n_{i}$ is the number of samples in class $i$, $\mu_{i}$ is the mean of class $i$, and $\mu$ is the overall mean of the data. By solving the generalized eigenvalue problem for the matrix $S_{W}^{-1}S_{B}$, LDA identifies the optimal projection direction that maximizes class separability. This method is particularly useful in scenarios where the dimensionality of the data is high relative to the number of samples, as it reduces the feature space while preserving class discriminatory information.

**4- Logistic Regression (LR)**

LR is a statistical method used for binary classification that estimates the probability of a categorical dependent variable based on one or more predictor variables. The core of LR is the logistic function which maps any real-valued number into (0, 1) interval, making it suitable for modeling probabilities. The logistic function is defined as:

$$\pi\left( X \right)= \frac{1}{1+e^{-(\beta_{0}+\beta_{1}X_{1}+\ldots+\beta_{k}X_{k})}}$$

where $\pi\left( X \right)$ represents the probability that dependent variable equals one given the predictors $X_{1}, X_{2}, \ldots, X_{k}$, and $\beta_{0}, \beta_{1}, \ldots, \beta_{k}$ are the coefficients of the model. The odds of the event can be expressed as:

$$\frac{\pi}{1+\pi}= \sum_{i=1}^{n} \left[ y_{i}\log\left( \pi_{i} \right)+(1-y_{i})\log(1-\pi_{i}) \right]$$

where $y_{i}$ is the observed outcome for each observation. LR is particularly useful where the response a binary variable of multiple input features.

**5- Random forest (RF)**

RF is a powerful ensemble learning algorithm used for both classification and regression. It operates by constructing a multiple of decision trees during training and outputting the mode or mean of their predictions. The algorithm utilizes a technique known as bootstrap aggregating or bagging, where multiple subsets of training data are sampled with replacement to create each split. This enhances model robustness and reduces overfitting. The final prediction for classification can be described as:

$$\hat{y}=mode \left( T_{1}\left( x \right), T_{2}\left( x \right), \ldots, T_{N}\left( x \right) \right)$$

where $T_{i}\left( x \right)$ is the prediction from the $i^{th}$ tree and $N$ is the total number of trees in the forest. The strength of RF lies in its ability to handle large datasets with higher dimensionality while maintaining accuracy and interpretability. By aggregating predictions from multiple trees, RF mitigates the risk of overfitting that can occur with individual decision trees, making it robust choice for complex data scenarios.

**6- Naïve Bayes (NB)**

NB is a family of probabilistic algorithms based on Bayes’ theorem, which is particularly effective for large datasets and text classification purposes. The main principle of NB is the assumption of conditional independence among features, meaning that a particular feature in a class is independent of the other feature. This simplifies the computation of the posterior probability, allowing for efficient classification. Given a dataset with features $X=\left( x_{1}, x_{2}, \ldots, x_{n} \right)$ and a class variable $Y$, the posterior probability can be expressed using Bayes’ theorem as:

$$P\left( X | Y \right)=P\left( x_{1} | Y \right)P\left( x_{2} | Y \right)\ldots P\left( x_{n} | Y \right)$$

Thus, the posterior probability becomes:

$$P\left( Y | X \right)\propto P\left( Y \right)\prod_{i=1}^{n} P\left( x_{i} | Y \right)$$

In practice, NB classifiers can be implemented with various distributions for continuous and categorical data. For continuous features, it often assumes a Gaussian distribution, estimating parameters such as mean and variance. NB performs well in many applications due the simplicity and efficiency.
